# Supplementary figures and images for: Identification of secreted proteins by comparison of protein abundance in conditioned media and cell lysates
Source: Anal Biochem. Author manuscript; Available in PMC 2022 Dec 16. (PMC9756135; doi:10.1016/j.ab.2022.114846)

Lysate abundance (BFA vs. Control)

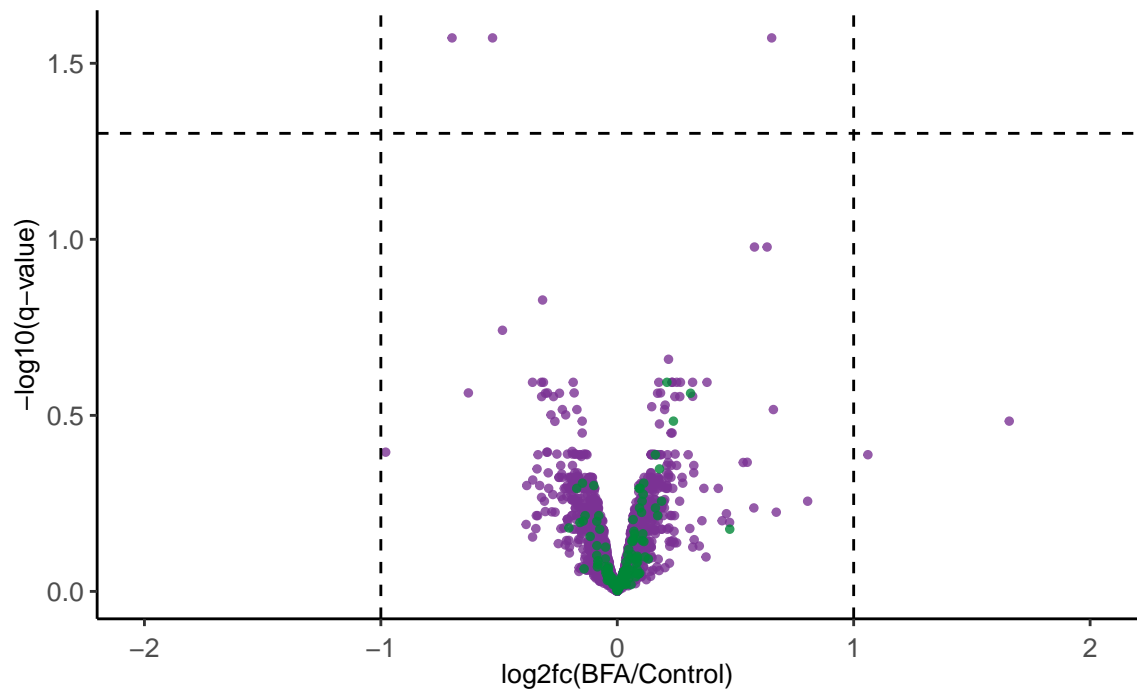

Media abundance (BFA vs. Control)

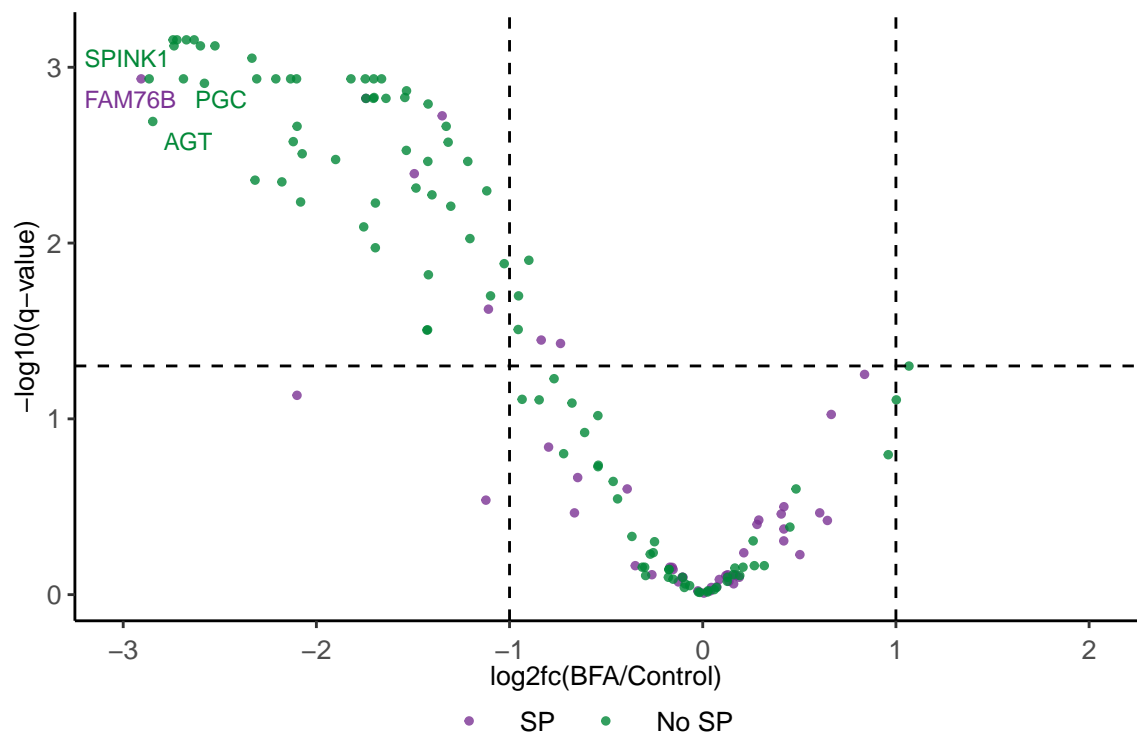

Supplement: Suppl Figs [file NIHMS1856908-supplement-Suppl_Figs.pdf]
